# Ovarian mucinous cystic tumor of borderline malignancy with a mural nodule of anaplastic spindle cell carcinoma: a case report

**DOI:** 10.1186/1757-2215-6-86

**Published:** 2013-12-05

**Authors:** Hitoshi Yamazaki, Akiyo Matsuzawa, Takashi Shoda, Hiroyoshi Iguchi, Noriyuki Kyushima

**Affiliations:** 1 Department of Pathology, Kitasato University, Sagamihara, Japan; 2 Department of Gynecology & Obstetrics, Kitasato University, Sagamihara, Japan; 3 Department of Radiology, Kitasato University, Sagamihara, Japan

**Keywords:** Ovarian mucinous cystic tumor of borderline malignancy, Sarcoma-like mural nodule (SLMN), Sarcomatous, Anaplastic spindle cell carcinoma, Mature cystic teratoma, Mucinous cystic neoplasm of the pancreas, Immunohistochemical, Case report

## Abstract

Ovarian cystic tumors with a mural nodule are a rare entity. We report a case of a mural nodule of anaplastic spindle cell carcinoma in an ovarian mucinous cystic tumor of borderline malignancy. The patient was a 45-years-old Japanese woman who presented with an ovarian cyst. She suffered from mature cystic teratoma of both ovaries 9 years before the present history. Image analysis and laboratory data showing a high serum CA19-9 level suggested ovarian malignancy. She underwent bilateral salpingo-oophorectomy with hysterectomy and omentectomy. There was a mural nodule in the ovarian mucinous cystic lesion. Microscopically, the nodule was composed of spindle-shaped cells with severe nuclear atypia. Immunohistochemical analysis allowed the cells to be categorized as anaplastic spindle cell carcinoma. Fifteen months after the operation the patient is alive without any clinical findings of tumor recurrence. To the best of our knowledge in the English literature, this is the first report of a mural nodule of an anaplastic spindle cell carcinoma within an ovarian mucinous cystic borderline tumor harboring previously confirmed cystic teratoma.

## Background

A mural nodule in an ovarian neoplasm is a rare entity with an incidence between 2 to 5 per million. The mural nodules are histologically divided into a wide variety from benign to malignant lesions. There are reported cases with a variety of mural nodules, such as anaplastic spindle cell carcinoma, carcinosarcoma, sarcoma-like mural nodule, sarcoma, and some other benign neoplasms [1-4]. Recent immunohistochemical analysis could elucidate the detailed histopathological characteristics of these nodules. The background ovarian neoplasm is cystic tumor and it is divided into two types, mucinous cystic tumor and serous cystic tumor. However, the former accounts for the majority of cases and the latter the minority. Mural nodules of benign neoplasms in serous cystic tumors are extremely rare [5,6]. Mucinous cystic neoplasms in other organs can also coexist with secondary tumors of the other histological type within the stroma [7-13]. The subject of this report was a mural nodule with features of anaplastic spindle cell carcinoma arising in an ovarian mucinous cystic tumor of borderline malignancy. Here, we emphasize the possible presence of totipotent stem cells in the stroma of mucinous tumors.

## Case presentation

**Figure 1:**
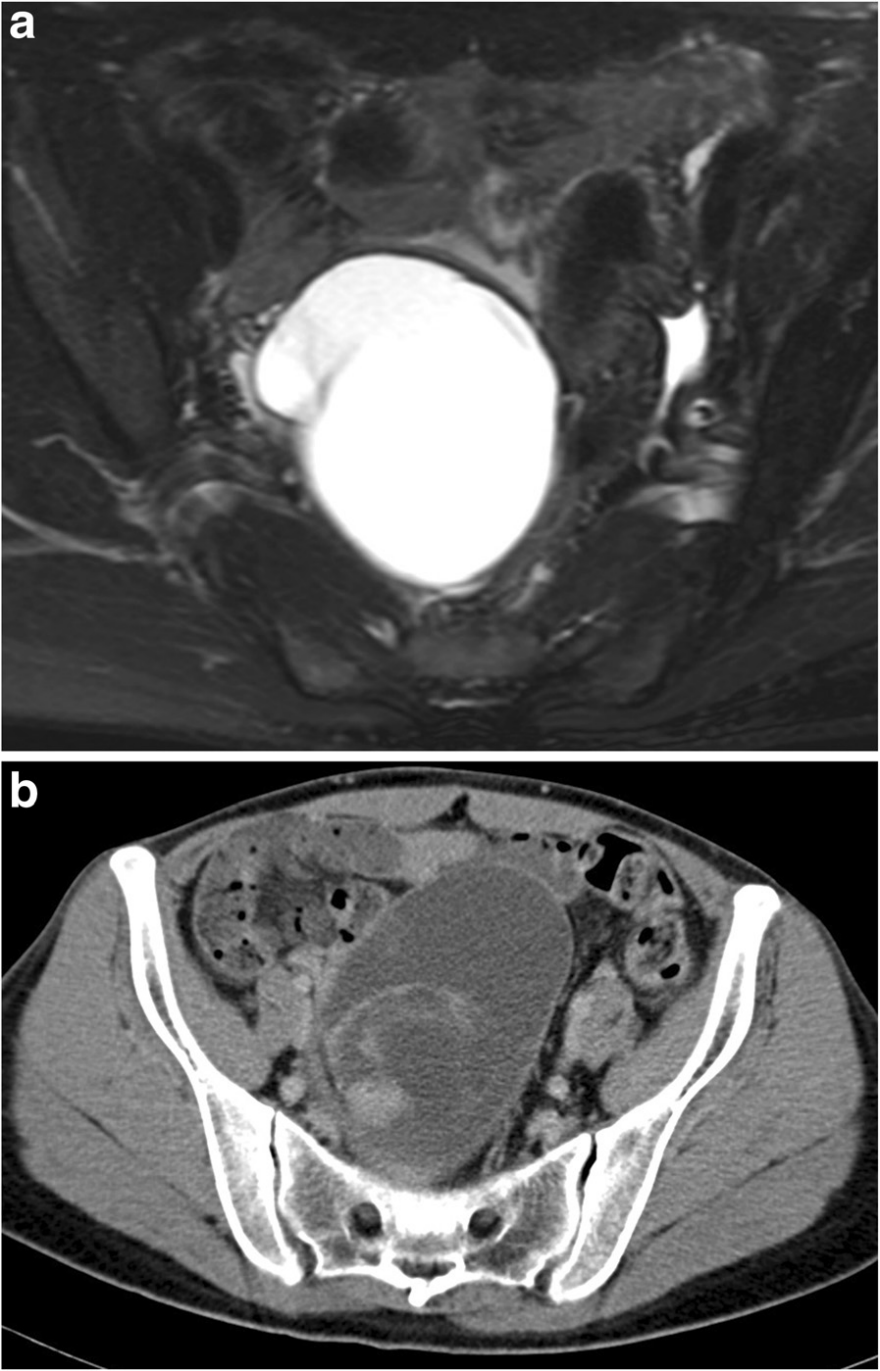
**Image analysis. a)** Gadrinium-enhanced T1 weighted magnetic resonance imaging shows a multilocular cystic lesion in the right ovary. **b)** Enhanced computed tomography shows an intraabdominal mass lesion occupying the pelvic cavity. Intracystic irregular enhancement was focally noted.

**Figure 2:**
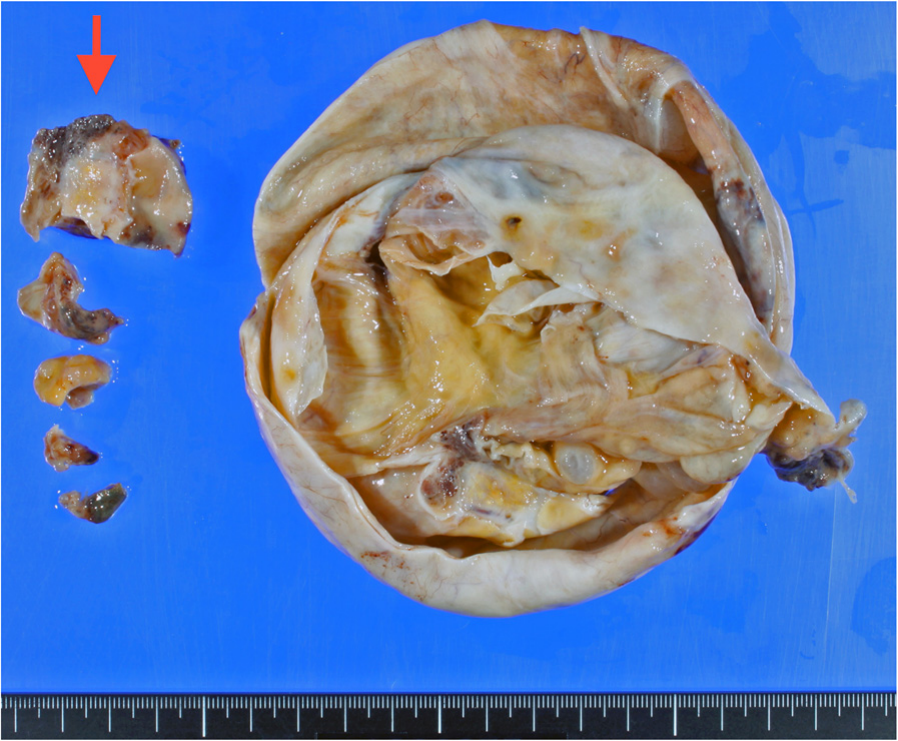
**Gross findings.** The inner surface of the main cystic cavity exhibits some thickening of the cyst wall. Red arrow indicates 5 pieces of the resected mural nodule, the largest of which was submitted for frozen sectioning at surgery. The others were totally embedded in paraffin and histologically analyzed.

**Figure 3:**
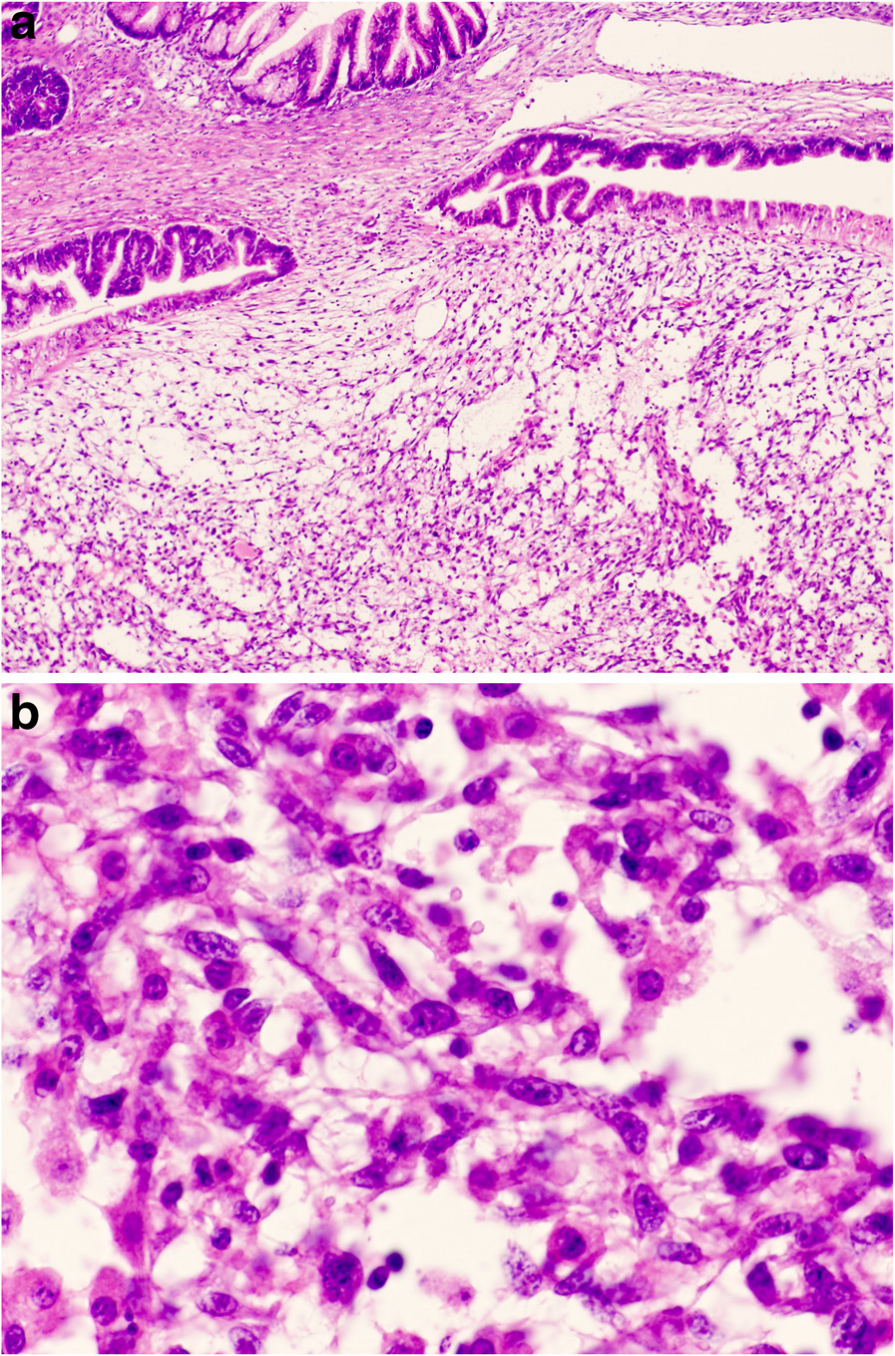
**Microscopic findings. a)** Hematoxylin and eosin-stained section shows the boundary between the cystic borderline tumor and the mural nodule (x 10). **b)** Higher magnification reveals the mural nodule is made up of sarcomatoid spindle cells (x 200).

**Figure 4:**
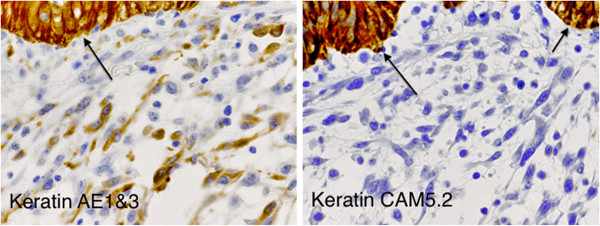
**Immunohistochemical analysis.** The arrow indicates the cystic borderline tumor cells, which are positive for both keratin AE1&3 and keratin CAM5.2. In contrast, the sarcomatoid spindle cells of the mural nodule are positive for keratin AE1&3, but negative for keratin CAM5.2 (x 100).

We present a rare case of ovarian mucinous cystic tumor of borderline malignancy with mural nodules of an anaplastic spindle cell carcinoma. A 45-year-old Japanese woman presented at our hospital following discovery of a left ovarian cyst during a periodic medical examination. She had presented 9 years earlier with ovarian cysts on both ovaries and these were diagnosed as ovarian mature cystic teratomas following ovarian cystectomy. Magnetic resonance imaging (MRI) revealed a right ovarian multilocular cyst of 70 × 70 × 64 mm (Figure 1a). One year later, marked enlargement of the right ovarian cyst (120 × 95 × 80 mm) was shown by an enhanced computed tomography scan of the abdomen (Figure 1b) and the serum level of CA19-9 increased up to 614 ng/ml. Intracystic irregular enhancement was focally noted. There was a strong suspicion of malignant transformation of the ovarian cyst. The patient underwent bilateral salpingo-oophorectomy with hysterectomy and omentectomy. At laparotomy, there were a few yellowish ascites in the peritoneal cavity. The resected ovarian cyst contained non-hemorrhagic mucinous fluid in multiloculated cavities. On gross examination, the inner surface of the cyst was mostly smooth with areas of irregular thickening of the cyst wall, which protruded into the cystic cavity (Figure 2). A representative specimen taken from a thickening was submitted for histological examination (frozen sections). The tentative diagnosis was sarcomatous elements in the cyst wall. The submitted specimen after frozen section analysis was artificially fragmented into pieces, the largest of which was whitish, relatively firm and 36 × 27 mm in size (Figure 2). Permanent sections after fixation by 10% formaldehyde revealed papillary growth of mucin- secreting lining cells in the inner surface of the main cystic lesion. The cells occasionally invaded the superficial part of the cyst wall (Figure 3a) and associated mild cytological atypia was observed. However, no aggressively deep invasion of the cells was observed in the cyst wall. These features were categorized as a mucinous cystic tumor of borderline malignancy. On the other hand, the thickening of the cyst wall mainly consisted of monotonously proliferating mononuclear spindle cells with severe nuclear atypia (Figure 3b). The mitotic index on average was about 4/10 high power fields (x 400). Relatively hypervascular stroma associated with endothelial proliferation was present at the periphery, at which the spindle cells diffusely invaded. There was no transition zone between the mucin-secreting lining cells and the spindle cells. Consequently, a so-called ‘collision tumor’ like appearance was observed. From these findings, we diagnosed the thickening as a mural nodule. Immunohistochemically, the mucin-secreting lining cells of the inner surface showed positive stainability for CA19-9 and some epithelial markers such as keratin AE1&3 and keratin CAM5.2 (Figure 4). However, the spindle cells showed positive stainability for keratin AE1&3 and were negative for CA19-9 and keratin CAM5.2 (Figure 4). Both cells showed negative staining for Inhibinα, Estrogen receptor, Progesterone receptor, CD10, Calretinin, Smooth muscle actin and S-100. The Ki-67 index of the spindle cells was about 40%. Based on these data, the spindle cells, the major components of the mural nodule, were characterized as an anaplastic spindle cell carcinoma. We then determined the final pathological diagnosis of ovarian mucinous cystic tumor of borderline malignancy with a mural nodule of anaplastic spindle cell carcinoma. Fifteen months after the operation, the patient is alive without any clinical findings of tumor recurrence.

## Discussion

Ovarian tumors with a mural nodule are rare. Prat and Scully first introduced cases of mural nodules in a mucinous cystic tumor [14]. The background tumors of the mural nodule are in many cases divided into two categories, such as mucinous cystic tumor and serous cystic tumor. However, the former is more frequent than the latter [3,5,6]. In contrast with the distinct division of the background cystic tumors, many histological types of mural nodules have been reported. The nodules may be reactive or neoplastic, which are categorized as epithelial tumors, non-epithelial tumors and mixed tumors [15,16]. Furthermore, the nodules are benign [1-3] or malignant. Because recent immunohistochemical analysis could elucidate that the tumor cells of the mural nodule possess epithelial and/or non-epithelial characteristics, the name sarcoma-like mural nodule (SLMN) alone may be insufficient after detailed histological evaluation.

The etiology of the mural nodules is unclear. In addition to ovarian cancer, mural nodules associated with mucinous cystic neoplasm (MCN) of the pancreas [7-9] and gallbladder [10] have been reported. In general, the stroma neighboring the pancreatic MCN exhibits ovarian stroma-like properties, which are characterized by immunohistochemically positive stainability for Estrogen receptor, Progesterone receptor and Inhibin. The mural nodules associated with pancreatic MCN also include various histological tumors such as anaplastic carcinoma [8,12], malignant fibrous histiocytoma [11] and other sarcomatous lesions [7,9]. The wide histological variety of nodules types is one of the most interesting phenomena. Park TC reported a case of a mural nodule producing granulocyte colony stimulating factor (G-CSF) [17]. An ovarian carcinoma cell line established from anaplastic carcinoma produced granulocyte colony stimulating factor (G-CSF) [18]. These findings suggest that the mural nodules are derived from potential pluripotent cells in the stroma of MCN and that the etiology of some types of mural nodules is related to the presence of mucin-producing epithelial neoplasms. From another standpoint, so-called ‘collision tumors’ may be one of the most likely explanations for the presence of mural nodules [4,13]. Van den Berg et al. analyzed three cases of pancreatic mucinous cystic neoplasms with sarcomatous stroma using molecular biological techniques [13]. They indicated that a mucinous tumor and the sarcomatous components were clonally different from each other.

In our case, mature cystic teratoma was previously found. Some teratomas may have some relation to the origin of mural nodules and malignant transformation of teratoma is sometimes encountered [19]. Teratoma is a common tumor in the ovaries and is believed to be derived from pluripotent cells. We are very interested in the previous presence of teratoma and a possible link to mucinous cystic tumors with mural nodules. However, there is no direct evidence of a relationship between teratoma and the mural nodules of ovarian MCN.

Provenza said FIGO stage 1a mucinous tumors had a favorable prognosis [20]. Poor prognosis in some cases depended on the malignant potential of the mural nodules [5,14,21]. However, good clinical outcome was reported in other studies [6,22-24]. Chan YF et al. emphasized the importance of adjuvant chemotherapy in postoperative management [25]. In our case, postoperative adjuvant chemotherapy was not performed as the patient was categorized as FIGO stage 1a. She is alive without any clinical findings of tumor recurrence fifteen months after the operation. Careful follow-up is now underway as anaplastic carcinomas sometimes have unfavorable prognoses in the reported cases.

In summary, we report an extremely rare case of a mural nodule of anaplastic spindle cell carcinoma arising in an ovarian mucinous cystic tumor of borderline malignancy. Furthermore, the presence of a previously confirmed cystic teratoma in the affected ovary was established. We think the etiology of mural nodules may be related to potentially totipotent stem cells present in the stroma of ovarian mucinous cystic tumors.

## Conclusions

According to the reported English literature, MCN of the ovary and MCN of other organs with ovarian stroma appear to lead to a tumorigenic environment in the stroma. To the best of our knowledge, this is the first report of a mural nodule of anaplastic spindle cell carcinoma in an ovarian mucinous cystic borderline tumor harboring a previously confirmed cystic teratoma.

## Consent

Written informed consent was obtained from the patient for publication of this case report and accompanying images. A copy of the written consent is available for review by the Editor-in-Chief of Journal of Ovarian Research.

## Competing interests

The authors declare that they have no competing interests.

## Authors’ contributions

HY was the pathologist for histopathological diagnosis of the case, designed the study and drafted the manuscript. AM, TS and NK were the gynecologists who performed surgery and took all clinical precautions. HI was a radiologist and did the image analysis. All authors read and approved the final manuscript.
